# Surgical Site Infections and Other Postoperative Complications following Prophylactic Anticoagulation in Total Joint Arthroplasty

**DOI:** 10.1371/journal.pone.0091755

**Published:** 2014-04-09

**Authors:** Zhong Wang, Frederick A. Anderson, Michael Ward, Timothy Bhattacharyya

**Affiliations:** 1 Clinical Trials and Outcomes Branch, Intramural Research Program, National Institute of Arthritis and Musculoskeletal and Skin Diseases, National Institutes of Health, Bethesda, Maryland; 2 Center for Outcomes Research, University of Massachusetts Medical School, Worcester, Massachusetts; Maastricht University Medical Center, Netherlands

## Abstract

**Background:**

Anticoagulants reduce the risk of venous thromboembolism (VTE) after total joint replacement. However, concern remains that pharmacologic VTE prophylaxis can lead to bleeding, which may impact on postoperative complications such as infections and reoperations.

**Methods and Findings:**

From the Global Orthopedic Registry (GLORY), we reviewed 3,755 patients in US who elected for primary total hip or knee arthroplasty, received either warfarin or low molecular weight heparin (LMWH) as VTE prophylactics, and had up-to-90-day follow-up after discharge. We compared incidence rates of VTE, infections and other complications between LMWH and warfarin groups, and used multivariate analyses with propensity score weighting to generate the odds ratio (OR). Patients receiving LMWH tended to be older and higher in the American Society of Anesthesiologists grade scores. In contrast, warfarin was used more frequently for hip arthroplasty with longer duration among patients with more pre-existing comorbidity (all P<0.02). A weight variable was created with propensity score to account for differences in covariate distributions. Propensity score-weighted analyses showed no differences in VTE complications. However, compared to warfarin, LMWH was associated with significantly higher rates of bleeding (6.2% vs. 2.1%; OR = 3.82, 95% confidence interval [CI], 2.64 to 5.52), blood transfusion (29.4% vs. 22.0%; OR = 1.75, 95% CI, 1.51 to 2.04), reoperations (2.4% vs. 1.3%; OR = 1.77, 95% CI, 1.07 to 2.93) and infections (1.6% vs. 0.6%; OR = 2.79, 95% CI, 1.42 to 5.45). Similar results were obtained from compliant uses of warfarin (26%) and LMWH (62%) according to clinical guidelines. While surgical site infections were mostly superficial, current study was underpowered to compare incidence rates of deep infections (<1.0%).

**Conclusions:**

Surgical site infections and reoperations in 3 months following primary total joint arthroplasty may be associated with anticoagulant use that exhibited higher bleeding risk. Long-term complications and deep wound infections remain to be studied.

## Introduction

It is well established that anticoagulant prophylaxis reduces symptomatic deep vein thrombosis (DVT) or venous thromboembolism (VTE) following elective total joint arthroplasty. Numerous chemoprophylactic regimens have been incorporated into evidence based guidelines [1], [2]. However, there remains an intrinsic balance between preventing VTE through anticoagulation and avoiding excess bleeding due to anticoagulant use. Surgeons have in the past expressed great concern that postoperative bleeding could lead to surgical site complications [3]. Surgical site complications such as infections represent potentially serious complications [4] that delay patient recovery and increase the burden to the healthcare system [5] and remain as one of the main reasons for revision surgery [6]. Excess bleeding associated with prophylactic use of anticoagulants could contribute to complications such as oozing [7], hematoma formation and wound drainage [8]. Although previous single site studies suggested an association between anticoagulant prophylaxis and postoperative infections [9]–[11], there have been no multi-center studies that addressed the associations between VTE prophylaxis and surgical site infections [12].

In this study, we analyzed data from the Global Orthopedic Registry (GLORY), an international registry that collected data from surgeons who used different VTE prophylactics for patients undergoing primary elective total hip and knee arthroplasty [13]. We compared the incidence rates of postoperative complications associated with two most common prophylactic treatment regimens in the United States, i.e., low molecular weight heparin (LMWH) and warfarin, which have been shown to differ in their risk profiles for bleeding [14].

## Methods

### Ethics Statement

The study was exempted by the institutional review boards at the National Institutes of Health and no informed consent was deemed necessary for this study, although individual consent had been obtained from patients who participated in GLORY.

### Data Sources and Study Population

**Figure 1:**
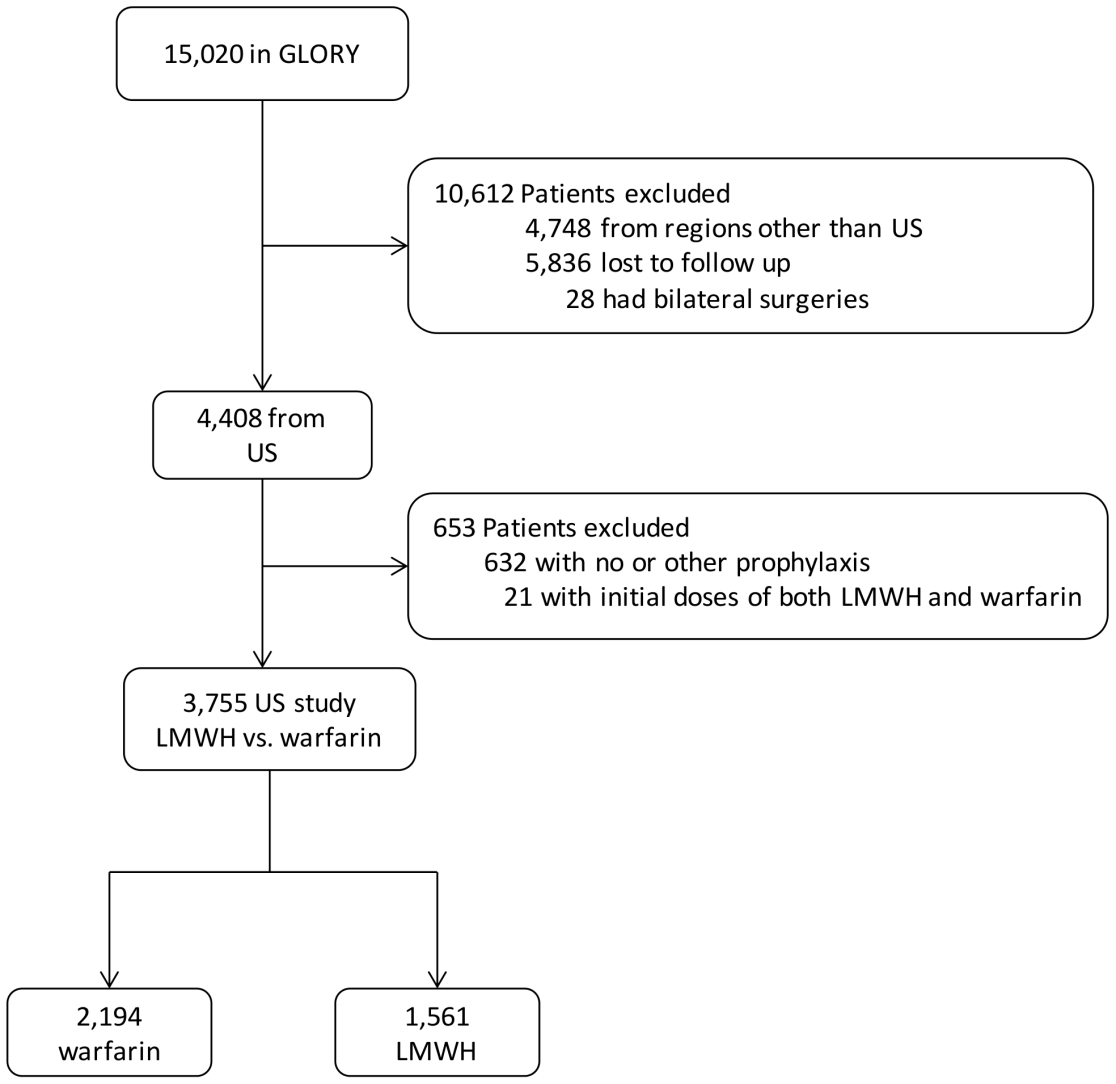
Study cohort. Note that 84.7% of patients who received warfarin and 93.3% of those with LMWH also received elastic stockings and/or intermittent pneumatic compression devices. There were 30 patients of US patients who did not have record for prophylaxis, and 159 of them receiving mechanical prophylaxis only. GLORY: Global Orthopedic Registry. LMWH, low molecular weight heparin.

The study was initiated following the completion of GLORY and publications of its findings [15]–[18]. The GLORY registry was designed to monitor a wide range of practices, complications and outcomes. Briefly, 156 orthopedic surgeons from 100 hospitals in 13 countries prospectively collected information on standard case report forms from the first 10 cases of elective hip or knee arthroplasty patients each month from 2001 to 2004. Data were centrally managed about their demographics, treatment regimen and monitoring of complications during in-hospital stays, 3-month and 12-month follow-ups. We only included patients with up to 3 months of follow up for this analysis, due to excessive lost-to-follow-up at 12-month follow-up. In addition, we chose the US region because LMWH and warfarin were utilized as two of the most prevalent forms of pharmacologic prophylaxes in US (Figure 1), whereas most patients from Europe received LMWH. We did not include the naïve control group who did not have any record for prophylaxis (n = 30), or those who only got mechanical prophylaxis (n = 159).

### Outcomes

The primary outcomes were clinician-reported symptomatic VTE during hospitalization or within 3 months after hospital discharge. Surgical site infections or reoperations were reported respectively as wound infections or surgical procedures involving incision, within 90-day period following surgery. VTE included symptomatic deep vein thrombosis (confirmed by venography, duplex ultrasound, or other objective method of diagnosis) or symptomatic pulmonary embolism (confirmed by lung scan, CT, pulmonary angiogram, or other objective method of diagnosis). Other outcomes included length of hospital stay for the primary procedure, bleeding, blood transfusion, the volume of blood transfused and miscellaneous complications. For bleeding, we included those cases with two or more units of blood transfused during surgery, plus bleeding-related complications such as reoperation due to bleeding, delayed hospital discharge due to bleeding, hematoma requiring evacuation, epidural hematoma, gastrointestinal bleeding, GI bleed, and hemorrhage requiring hospital readmission.

### Statistical Analysis

Baseline characteristics of the patients were compared between treatment groups with the Pearson chi-square test. As most of the outcomes were binary (yes/no), we used multivariate logistic regressions with choice of prophylaxis as the main covariate in addition to the following variables: age, sex, body-mass index (BMI), year of surgery, type of surgery (hip/knee), antibiotic use, length of surgery, co-morbid conditions, and the American society of Anesthesiologists (ASA) score.

Since these prophylaxis groups were not randomly assigned but different with respect to patients' demographic and clinical characteristics, we balanced the groups with propensity score adjustment. Propensity scoring is a well-established statistical method that controls for selection bias in observational studies by using a weighted score to balance the two cohorts [19]–[21]. Propensity scores were generated using multivariate logistic regression to calculate probability of receiving warfarin vs LMWH, based on patient characteristics including age, sex, BMI, year of surgery, type of surgery, antibiotic use, length of surgery, co-morbid conditions, and ASA score.

As is common in multicenter registry studies, a number of patients had missing values for variables such as weight or ASA score. Missing values would have substantially reduced our sample size. Thus prior to the propensity score weighting, multiple imputation was used to impute variables with missing values [22], [23]. Multiple imputation was implemented not only to reduce estimate bias associated with complete case analysis, but also to improve the performance of propensity scores. To implement the multiple imputation, we used IVEware version 2.0 (Ann Arbor, MI) to generate 5 datasets with the same number of observations as the original dataset [24]. Propensity scores were generated for each dataset and effect size was estimated per dataset. To summarize the data, Rubin's rule was used to generate the final statistics [22], [25]. We performed additional analyses by setting missing to a separate category in the original dataset and results were similar. It should be noted that missing values were imputed only for covariates, not for outcomes or exposure variables.

All significance tests were conducted at two-sided level of 0.05. Since testing for association between prophylaxes and numerous complications was planned a priori, we did not adjust α level for multiple comparisons. Due to limited sample size, we only conducted significance tests for those comparison groups with minimal difference of 20% and one of rates at least over 1%. The statistical analysis was conducted using SAS 9.3 (SAS Institute Inc, Cary, North Carolina).

## Results

### Cohort Characteristics

From the US study population, 1,508 and 2,247 patients underwent hip and knee arthroplasty, respectively. Among these patients, 2,194 initiated prophylaxis with warfarin whereas 1,561 received prophylaxis with LMWH. For prophylaxis, warfarin was taken either preoperatively (40.0%) or within 24 hours postoperatively (60.0%), whereas LMWH was administered in 81% of cases from 7 to 36 hours following surgery. It should be noted that 85% of patients who received warfarin and 93% of those with LMWH also received elastic stockings and/or intermittent pneumatic compression devices.

Table 1 shows selected baseline characteristics of the study population. Significant differences were observed among all the variables related to the patient demographic and clinical characteristics except for sex and BMI. Patients treated with LMWH tended to be older and higher in the American Society of Anesthesiologists grade scores. In contrast, warfarin was used among patients with more pre-existing conditions (all P<0.02). We used propensity score weighting to control for the differences in covariate distributions as shown in Table 1.

**Table 1:** Baseline Characteristics of the US Patients from the Global Orthopedic Registry (GLORY, N = 3,755).

	Unadjusted			Adjusted with Propensity Score Weighting		
	LMWH (N = 1,561)	Warfarin (N = 2,194)	P Value	LMWH (N = 1,561)	Warfarin (N = 2,194)	P Value
Year of surgery			0.006			0.99
2001	12.9	14.0		13.8	13.8	
2002	37.9	33.4		35.2	35.1	
2003	32.1	36.8		34.4	34.7	
2004	17.1	15.8		16.6	16.4	
Age in years			0.002			0.99
18–54	13.7	18.2		16.3	16.2	
55–64	23.9	21.8		22.8	22.8	
65–74	34.3	33.9		33.6	33.9	
75+	28.1	26.1		27.3	27.1	
Joint			<0.001			0.82
Hip	31.1	45.9		40.4	40.0	
Knee	68.9	54.2		59.6	60.0	
ASA scores			0.002			0.86
No chronic conditions	12.7	16.1		15.6	15.0	
Mild chronic conditions	57.5	58.3		57.9	58.2	
Severe or moribound	29.8	25.6		26.4	26.8	
Sex			0.67			0.89
Male	41.1	41.8		41.7	41.5	
Female	58.9	58.2		58.3	58.5	
Body mass index (BMI)			0.41			0.99
Under Weight	0.5	0.6		0.6	0.6	
Norm Weight	15.2	16.2		16.3	16.3	
Over Weight	33.7	35.6		34.5	34.6	
Obese	50.6	47.6		48.6	48.5	
Length of surgery			<0.001			0.86
<2 Hours	91.6	81.4		86.7	87.1	
2–4 Hours	8.3	18.3		13.1	12.8	
>4 Hours†	0.1	0.3		0.2	0.1	
Prior conditions			<0.001			0.75
No	86.4	81.0		82.7	83.2	
Yes	13.6	19.0		17.3	16.8	
Antibiotics use			0.004			0.87
None	0.5	1.6		1.1	1.1	
Prophylaxis only	97.9	96.4		96.6	96.9	
Additional Indication	1.7	2.0		2.3	2.0	

ASA, American society of Anesthesiologists; LMWH, low molecular weight heparin.

### Bivariate and Multivariate Analyses

Since the test results were similar between bivariate and multivariate analyses, we only show the P values following multivariate analyses with propensity-score weighting. As shown in Table 2, the lengths of hospital stay were not significantly different between two groups (P = 0.40) and the overall risks of general medical complications were similar between LMWH and warfarin groups (LMWH vs. warfarin: 3.1 vs 2.6%; OR, 1.41; 95% CI, 0.96 to 2.07). Similarly, the risks of symptomatic VTE were comparable between the two groups (LMWH vs. warfarin: 1.5 vs 0.9%; OR, 1.72; 95% CI, 0.93 to 3.17) (Table 2).

**Table 2:** Comparison of Outcomes from the US Study Population of the Global Orthopedic Registry (GLORY).

	LMWH†(N = 1,561)	Warfarin† (N = 2,194)	Odds Ratio*(95% CI)	P Value
Length of stay, median (IQR)	3 (3–5)	4 (3–4)	0.98 (0.93, 1.03)	0.40
General complications	48 (3.1)	58 (2.6)	1.41 (0.96, 2.07)	0.08
Cardiac	9 (0.6)	11 (0.5)		
Medical	16 (1.0)	19 (0.9)		
Surgical	25 (1.6)	31 (1.4)		
Symptomatic VTE	23 (1.5)	19 (0.9)	1.72 (0.93, 3.17)	0.08
Blood transfusion	459 (29.4)	483 (22.0)	1.75 (1.51, 2.04)	<.001
Volume (ml), mean (SD)	614 (371)	524 (227)		<.001
Bleeding	97 (6.2)	45 (2.1)	3.82 (2.64, 5.52)	<.001
Bleeding Complications	13 (0.8)	6 (0.3)	2.95 (1.10, 7.89)	0.03
Surgical site infection	25 (1.6)	13 (0.6)	2.79 (1.42, 5.45)	0.003
Superficial infection	20 (1.3)	9 (0.4)	3.47 (1.53, 7.84)	0.003
Deep infection	5 (0.4)	4 (0.2)		
Reoperation	38 (2.3)	28 (1.3)	1.77 (1.07, 2.93)	0.03
Due to infection	11 (0.7)	11 (0.5)		

LMWH: low molecular weight heparin; IQR: inter quartile range; SD: standard deviation; CI, confidence interval; VTE: venous thromboembolism.

*LMWH vs. Warfarin.

† Values are given as the number of patients with the percentage in parentheses, unless otherwise indicated.

However, compared to those with warfarin, patients treated with LMWH were more likely to receive blood transfusion (LMWH vs. warfarin: 29.4 vs 22.0%; OR, 1.75; 95% CI, 1.51 to 2.04) and had higher volume of blood transfusion (LMWH vs. warfarin: mean ± SD: 614±371 vs 524±227). They also had significantly higher risks for bleeding (LMWH vs. warfarin: 6.2 vs 2.1%; OR, 3.82; 95% CI, 2.64 to 5.52) and surgical site infections (LMWH vs. warfarin: 1.6 vs 0.6%; OR, 2.79; 95% CI, 1.42 to 5.45). Most of the surgical site infections in the LMWH group were deemed by the reporting surgeon as superficial (LMWH vs. warfarin: 1.3 vs 0.4%; OR, 3.47; 95% CI, 1.53 to 7.84). No significance tests were conducted in the rates of deep infections, per pre-specified testing rule.

In addition, patients treated with LMWH had higher rates of reoperation compared to those with warfarin (LMWH vs. warfarin: 2.3 vs 1.3%; OR, 1.77; 95% CI, 1.07 to 2.93). Reoperations due to infection were slightly higher in the LMWH group, but no significance test was done (LMWH vs. warfarin: 0.7 vs 0.5%). 21 out of 49 infections were treated with reoperations, wherein 9 out of 11 deep infections and 12 out of 38 superficial infections did.

### Subgroup Analyses

Using 2001 edition of American College of Chest Physicians (ACCP) guidelines for VTE prophylaxis, we limited the sample to patients who were compliant with the contemporaneous ACCP guidelines in terms of type, duration, starting time and dose of prophylactics. Based on whether a target international normalization ratio (INR) of 2.0–3.0 was achieved, only 26.2% of warfarin use was compliant (n = 575). In comparison, 62.3% use of LMWH was compliant based on the dosage and time window of commencement (at regular dosage either within 12 hours before the surgery, or 12–24 hours after surgery, or at half the usual dose within 4–6 hours after surgery and continuing with the usual dose on the following day, n = 973). As shown in Table 3, the incidence rates of infections within 3 months after discharge for LMWH sub group was 2.0%, compared to 0.4% in the warfarin subgroup (OR, 5.07; 95% CI, 1.30 to 19.77; P = 0.02). The risk of bleeding was significantly higher in LMWH group (OR, 3.98; 95% CI, 2.10 to 7.56; P<0.01) as was that of reoperation (OR, 3.42; 95% CI, 1.26 to 9.29; P = 0.02).

**Table 3:** Subgroup Analysis of Compliant Use from the US Study Population of the Global Orthopedic Registry (GLORY).

	LMWH†(N = 973)	Warfarin†(N = 575)	Odds Ratio*(95% CI)	P Value
Bleeding	66 (6.8)	16 (2.8)	3.98 (2.10, 7.56)	<.001
Bleeding Complications	6 (0.6)	1 (0.2)		
Surgical site infection*	20 (2.0)	3 (0.4)	5.07 (1.30, 19.77)	0.02
Superficial infection	17 (1.7)	2 (0.3)	5.63 (1.18, 26.99)	0.03
Deep infection	3 (0.4)	1 (0.1)		
Reoperation*	29 (3.0)	6 (0.9)	3.42 (1.26, 9.29)	0.02

LMWH: low molecular weight heparin; CI, confidence interval; VTE: venous thromboembolism.

*LMWH vs. Warfarin.

† Values are given as the number of patients with the percentage in parentheses.

## Discussion

In this registry based analysis, we observed that compared to warfarin, LMWH was associated with higher incidences of blood transfusions and bleeding. Concurrently, we also observed that LMWH was associated with higher rates of wound infections and reoperations. This is consistent with our hypothesis that patients treated with different anticoagulants with varying bleeding risk, would have significant difference in infections or other complications. However, the clinical significance of our observation is not clear as the majority of infections were deemed superficial with nearly half treated without reoperation. The limited number of bleeding episodes and deep infections prevented us from studying the involvement of bleeding and clinical significance of infections.

Several studies have noted that excessive anticoagulation was associated with prolonged wound drainage that is conducive for the development of infection. There seems to be a clinical balance between providing anticoagulation that prevents deep vein thrombosis and allowing the surgical wound to heal. Hematoma formation can result in wound drainage that can predispose patients towards infection [10], [11], [26]–[29]. Thus, the results of our study are expected based on these observations.

Though the GLORY registry is large and national in scope, we found two difficulties in analyzing the data. First, we found that the patients treated with LWMH formed a different cohort than warfarin patients. We used propensity score matching to balance the respective cohorts such that comparison could be allowed. Second, we found that a number of variables (such as duration of surgery) were missing from the registry. Restricting the analysis to only those cases with complete data would have degraded the sample size considerably and biased the results, so multiple imputation was used to address missing data [19]–[21]. We compared the distributions of imputed variables such as length of surgery among US surgeons with those of surgeons from Europe where data about the length of surgery was complete, and found that following imputation, the two distributions were similar (data not shown).

The incidence rate of symptomatic VTE in LMWH group was higher than that in Warfarin group, although the difference didn't reach statistical significance. We suspect that surgeons might pay more attention to the newly-introduced LMWH and be more on the lookout for VTE symptoms among patients treated with LMWH. However, the same could not be said about infections, because no link had been suspected between these two at that time. So there may be some ascertainment bias for the VTE outcomes, but not for infections. While we showed that LMWH use was similar in its effectiveness to prevent symptomatic VTE as warfarin, we observed a significant increase in surgical site infections in patients treated with LMWH. We attribute this to the fact that most GLORY patients from US received LMWH within 12–24 hours of surgery (as per ACCP guidelines). Thus anticoagulation is present immediately with a fresh postoperative wound and while patients are undergoing early rehabilitation, hindering the wound healing process and exposing patients to potential infectious agents. In fact, we observed in a separate analysis of GLORY data from Europe that timing of LMWH prophylaxis around surgical time was associated with significantly higher risk of infections (unpublished observation).It should be mentioned that more than half of these surgical site infections (28 of 49) were treated without reoperation. Clearly the line between a superficial and a deep infection is a gray line, most surgeons would be wary of any level of infection. Furthermore, the rarity of deep infections (0.29%) makes comparison of this outcome impossible in this study.

We observed an increase in reoperations in the LMWH group, but were not able to test if there was an increase in reoperations specifically due to infection. We attribute this to a limitation in registry data. In GLORY, the reason for reoperation was not always clearly delineated; 29 out 76 patients had reoperations without an attributable cause (data not shown). We acknowledge that in a clinical scenario, a patient may have a reoperation for multiple causes (e.g. dislocation in conjunction with wound drainage) and in un-adjudicated study setting, it is not always simple to attribute the cause of a reoperation. However, from the patient's perspective, any reoperation is a negative outcome and should be evaluated as we did.

Using ACCP guidelines, we found that compliance among patients treated with warfarin was much lower than that among patients with LMWH, presumably due to a preference by surgeons to limit the INR to levels below those recommended by clinical guidelines. This caused some concerns that our comparison between LMWH and warfarin may be problematic due to low dosage of warfarin. However, our analysis among subset of patients with compliant use of either agent clearly demonstrated the association with more infectious outcomes following LMWH treatment.

Our study has several limitations that may affect its internal and external validity. First, although this registry was designed for identifying functional outcomes and complications following hip or knee arthroplasty, it is mostly used for generating hypothesis, as well as providing information about real-world practices. Second, with prophylaxis choice not randomized, the difference we found in the infectious outcomes may not be attributable to the exposure we studied. Even after we used propensity score weighting to balance the difference between treatment groups, there remain other unknown or unobserved confounders such as hospital or surgeon procedure volume [30] and clinic/hospital urban/teaching status [5]. Third, due to high compliance with VTE prophylaxis, there are few patients in naïve control group from the registry, preventing us from constructing a placebo group. However, unadjusted analysis did show that baseline rate of infections in naïve control group was similar to that of warfarin group but less than that of LMWH group (unpublished observation). And finally, the sample size was enough for the main outcomes (bleeding and infections) but not for most subgroup analyses. The limited size of the study and voluntary nature of surgeon participation may render the study less generalizable.

Given that there are approximately 1 million major orthopedic procedures each year in US, doubling of the risk of infection from less than 1% to approximately 2% may present significant burden to the healthcare system. This investigation is consistent with the observed increase in the infection burden in US for hip and knee arthroplasty from 1998 to 2004 when the use of LMWH use became increasingly prevalent [5]. However, LMWH should not be abandoned for prophylactic use against venous thromboembolism. Instead, it should be used prudently as shown in clinical practices [31] and hospital settings [32]. The same prudence should be exercised for any new anticoagulant, as a recent study reported significant wound complications following administration of direct factor-Xa inhibitor as a stronger thrombotic agent compared to LMWH in lower limb arthroplasty [33].

In conclusion, the choice of thromboprophylaxis may be associated with significant higher incidences of surgical site infections and reoperations, in addition to bleeding. Postoperative surgical site infection should be assessed routinely in future clinical trials of new anticoagulants and registry for joint replacements, as this may impact both risk-benefit and cost-benefit evaluations of VTE prevention regimens following joint arthroplasty.
